# How Gender Shapes the Effects of Immediate Family Members’ Deaths on Adults’ Psychological Well-Being

**DOI:** 10.1093/geroni/igab046.352

**Published:** 2021-12-17

**Authors:** J Jill Suitor, Megan Gilligan, Catherine Stepniak, Yifei Hou, Robert Frase

**Affiliations:** 1 Purdue University, Purdue University, Indiana, United States; 2 Iowa State University, Iowa State University, Iowa, United States; 3 Purdue University, West Lafayette, Indiana, United States

## Abstract

The deaths of family members constitute one of the most serious negative life events experienced in adulthood. The impact of these losses on psychological well-being may differ considerably by the structural relationship between the deceased and the survivors, and by the genders of both family members; however, few studies have been able to explore these variations by generation, gender, and time since death. In this paper, we use mixed-methods data to explore how depressive symptoms are affected differentially in adulthood by the deaths of mothers, fathers, and siblings, as well as by the gender of survivors. We address these questions using data collected from approximately 600 adult children nested within 250 later-life families, in which approximately 55% experienced the death of at least one parent and 15% experienced the death of a sibling in the previous decade. Preliminary multilevel regression analyses showed that deaths of siblings predicted sisters’ but not brothers’ depressive symptoms. In the case of parents, only mothers’ deaths were found to predict daughters’ depressive symptoms, whereas neither parents’ deaths predicted sons’ well-being. Further, these patterns differed little by time since death. Qualitative data revealed that women were more likely to report that both their mothers’ and siblings’ deaths had led to higher conflict within the sibling network, which previous research has shown predicts psychological well-being. Taken together, these findings demonstrate the salient role of gender in shaping well-being in the face of events of deaths of parents and siblings in adulthood.

